# Mechanical bowel preparation and antibiotics in elective colorectal surgery: network meta-analysis

**DOI:** 10.1093/bjsopen/zrad040

**Published:** 2023-05-31

**Authors:** Jonavan Tan, Éanna J Ryan, Matthew G Davey, Fiachra T McHugh, Ben Creavin, Maria C Whelan, Michael E Kelly, Paul C Neary, Dara O Kavanagh, James M O’Riordan

**Affiliations:** Department of Colorectal Surgery, Tallaght University Hospital, Tallaght, Dublin, Ireland; Department of Colorectal Surgery, Tallaght University Hospital, Tallaght, Dublin, Ireland; Department of Surgery, Royal College of Surgeons in Ireland, Dublin, Ireland; Department of Colorectal Surgery, Tallaght University Hospital, Tallaght, Dublin, Ireland; Department of Colorectal Surgery, Tallaght University Hospital, Tallaght, Dublin, Ireland; Department of Colorectal Surgery, Tallaght University Hospital, Tallaght, Dublin, Ireland; Department of Colorectal Surgery, Tallaght University Hospital, Tallaght, Dublin, Ireland; Department of Colorectal Surgery, Tallaght University Hospital, Tallaght, Dublin, Ireland; School of Medicine, Trinity College Dublin, College Green, Dublin, Ireland; Department of Colorectal Surgery, Tallaght University Hospital, Tallaght, Dublin, Ireland; Department of Surgical Affairs, Royal College of Surgeons in Ireland, Dublin, Ireland; Department of Colorectal Surgery, Tallaght University Hospital, Tallaght, Dublin, Ireland; School of Medicine, Trinity College Dublin, College Green, Dublin, Ireland

## Abstract

**Background:**

The use of intravenous antibiotics at anaesthetic induction in colorectal surgery is the standard of care. However, the role of mechanical bowel preparation, enemas, and oral antibiotics in surgical site infection, anastomotic leak, and other perioperative outcomes remains controversial. The aim of this study was to determine the optimal preoperative bowel preparation strategy in elective colorectal surgery.

**Methods:**

A systematic review and network meta-analysis of RCTs was performed with searches from PubMed/MEDLINE, Scopus, Embase, and the Cochrane Central Register of Controlled Trials from inception to December 2022. Primary outcomes included surgical site infection and anastomotic leak. Secondary outcomes included 30-day mortality rate, ileus, length of stay, return to theatre, other infections, and side effects of antibiotic therapy or bowel preparation.

**Results:**

Sixty RCTs involving 16 314 patients were included in the final analysis: 3465 (21.2 per cent) had intravenous antibiotics alone, 5268 (32.3 per cent) had intravenous antibiotics + mechanical bowel preparation, 1710 (10.5 per cent) had intravenous antibiotics + oral antibiotics, 4183 (25.6 per cent) had intravenous antibiotics + oral antibiotics + mechanical bowel preparation, 262 (1.6 per cent) had intravenous antibiotics + enemas, and 1426 (8.7 per cent) had oral antibiotics + mechanical bowel preparation. With intravenous antibiotics as a baseline comparator, network meta-analysis demonstrated a significant reduction in total surgical site infection risk with intravenous antibiotics + oral antibiotics (OR 0.47 (95 per cent c.i. 0.32 to 0.68)) and intravenous antibiotics + oral antibiotics + mechanical bowel preparation (OR 0.55 (95 per cent c.i. 0.40 to 0.76)), whereas oral antibiotics + mechanical bowel preparation resulted in a higher surgical site infection rate compared with intravenous antibiotics alone (OR 1.84 (95 per cent c.i. 1.20 to 2.81)). Anastomotic leak rates were lower with intravenous antibiotics + oral antibiotics (OR 0.63 (95 per cent c.i. 0.44 to 0.90)) and intravenous antibiotics + oral antibiotics + mechanical bowel preparation (OR 0.62 (95 per cent c.i. 0.41 to 0.94)) compared with intravenous antibiotics alone. There was no significant difference in outcomes with mechanical bowel preparation in the absence of intravenous antibiotics and oral antibiotics in the main analysis.

**Conclusion:**

A bowel preparation strategy with intravenous antibiotics + oral antibiotics, with or without mechanical bowel preparation, should represent the standard of care for patients undergoing elective colorectal surgery.

## Teaser

A systematic review and network meta-analysis of RCTs was performed to determine the optimal preoperative bowel preparation strategy in elective colorectal surgery. Sixty RCTs involving 16 314 patients were included; network meta-analysis demonstrated a significant reduction in surgical site infection and anastomotic leak risk with strategies consisting of combined intravenous and oral antibiotics. Bowel preparation with intravenous and oral antibiotics, with or without mechanical bowel preparation, should represent the standard of care for patients undergoing elective colorectal surgery.

## Introduction

Infectious complications are common after colorectal surgery. The incidence of surgical site infection (SSI) is between 5.4 and 23.2 per cent^1^, comprising superficial and deep incisional SSIs as well as intra-abdominal abscess (IAA)/organ space infections, which occur in 7.9–11.5 per cent of cases^2,3^. The most feared complication is anastomotic leak (AL), which occurs in 2–10 per cent of cases, and more frequently in the setting of low rectal, complex inflammatory, and cancer resections^4,5^. Superficial SSIs incur a large burden on the healthcare system with increased utilization and costs^6,7^. IAA and AL represent significant morbidity that often requires further intervention, with an associated mortality rate as high as 16 per cent in the setting of AL^8,9^.

Routine prophylactic intravenous antibiotics (IAB) at induction of anaesthesia is the standard of care in elective colorectal surgery^10,11^, due to the observed reduction in SSI and other infectious complications. In contrast, ongoing debate exists regarding the role of mechanical bowel preparation (MBP) and oral antibiotics (OAB) in elective colorectal surgery. MBP theoretically allows for improved bowel handling, reduced faecal contamination and spillage, and reduced luminal pressure and bacterial load within the intestinal lumen. Moreover, OAB may further target the gastrointestinal bacterial flora, with direct exposure and further elimination of the colonic mucosa-related microflora^12,13^.

Early RCTs demonstrated evidence in support of the use of combined OAB + MBP for major colorectal surgery, with an observed reduction in SSI and AL rates^14–16^. However, concerns surrounding the effectiveness and morbidity of bowel preparation (including prolonged hospital admission, dehydration, electrolyte imbalances, *Clostridium difficile* infection (CDI), and patient discomfort) were subsequently raised^17–19^. Multiple RCTs in the early 2000s–2010s reported a marginal advantage for the use of preoperative MBP^20–27^, which was further supported in a Cochrane review performed by Güenaga *et al.*^28^ in 2011 and a subsequent meta-analysis performed by Cao *et al.*^29^ in 2012. Unfortunately, most of these studies failed to include OAB^20,21,23,25,26,30^, and MBP and OAB subsequently fell out of favour.

More recently, several large retrospective cohort studies of the American College of Surgeons (ACS) National Surgical Quality Improvement Program (NSQIP) colectomy database demonstrated significant reductions in the rates of SSI, AL, and post-operative ileus with OAB + MBP^31–33^. The ACS and Surgical Infection Society (2017)^34^, the American Society of Colon and Rectal Surgeons (2019)^35^, and the WHO^10^ guidelines recommend the use of OAB + MBP in elective colorectal surgery. However, there remains a large evidence–practice gap, with a recent international European multicentre audit in 2017 demonstrating low rates of utilization of this bowel preparation strategy, with only 16.8 per cent of patients receiving OAB + MBP *versus* 52.9 per cent receiving MBP^36^.

Given recent publication of further RCTs, the aim of this study was to review the prospective literature to evaluate the optimal bowel preparation to be used for elective colorectal surgery.

## Methods

A systematic review was performed according to the guidelines and recommendations of PRISMA^37^. Institutional review board approval was not required. The protocol for this meta-analysis was registered with the PROSPERO database (CRD42021287956).

### Search strategy

An electronic search for relevant publications was performed using the following sources: PubMed/MEDLINE, Scopus, Embase, and the Cochrane Central Register of Controlled Trials. The search headings and medical subject headings (MeSH) can be seen in *Fig. S1*. The search of the Cochrane Central Register of Controlled Trials, Embase, and Scopus databases was performed by combining the following search terms using the Boolean AND/OR operators: ‘colorectal surgery’, ‘surgery’, ‘colorectal’, ‘antibiotic’, and ‘bowel prep*’.

Publications were limited to those published in the English language. All titles were initially screened, and appropriate abstracts were reviewed. The reference section of each relevant publication and Google Scholar were also screened for other applicable publications. The function ‘related article’ in PubMed was also used to identify articles. In addition, clinicaltrials.gov was searched for proposed or ongoing trials. The last date of the search was 31 December 2022.

### Inclusion criteria

To be included in the analysis, the studies had to meet the following criteria: report on patients undergoing elective colorectal resection; compare the use of oral or systemic antibiotic use or MBP administration or both; report on surgical and clinical outcome measures mentioned below; have a clear research methodology and prospective randomization of patients; have the longest follow-up or the largest sample size when two or more studies were reported by the same institution; and be published in full-text format and in the English language.

### Exclusion criteria

Studies were excluded from the analysis if they failed to meet the above inclusion criteria.

### Outcomes of interest

The primary outcomes were total SSI and AL rates. SSI rates were subdivided into superficial SSI, deep incisional SSI, and IAA/organ space infection rates. Secondary outcomes included ileus, return to theatre, length of stay, urinary tract infection, respiratory tract infection, CDI, 30-day mortality rate, and side effects of antibiotic therapy or bowel preparation.

### Data extraction

Two authors (J.T. and F.T.M.) independently reviewed the literature according to the above predefined strategy and criteria. Each of these authors extracted the following data variables: titles and reference details (first author, journal, year, and country), study population characteristics (number in study, number treated by each approach, sex, and age), disease characteristics, type and approach of surgical intervention, and outcome data. All data were extracted independently into separate databases and compared at the end of the reviewing process to limit selection bias. Duplicates were removed, and any disparities were clarified. A third author (É.J.R.) independently reviewed the database and resolved any discrepancies.

### Statistical analysis

Descriptive statistics were used to report the characteristics of eligible trials. Binary data were compared using ORs. ORs were calculated using crude event data from the original articles to compare the efficacy of the various reconstructive strategies. Weighted-mean differences were calculated for continuous variables. If means and standard deviations were not available, estimates were derived from study data using the methods described by Hozo *et al*.^38^ and Luo *et al*.^39^.

Network meta-analysis (NMA) was conducted using the netmeta^40^ and Shiny^41^ packages for R. Effect sizes for the NMA are described with a 95 per cent c.i. Study heterogeneity was assessed via Cochrane Q score, *I*^2^ scores, and deviance information criterion between random- and fixed-effects models. A deviance information criterion difference of greater than three was deemed to indicate significant study heterogeneity and a random-effects model was used for all study arms^42^. Where appropriate, estimates of group means and standard deviations were calculated if the required data were available^38,39,43^. The authors plotted rank probabilities against the possible ranks for all competing treatments. The confidence in estimates of the outcome was assessed using Confidence in Network Meta-Analysis (CINeMA)^44^. Methodological assessment of included studies was undertaken by J.T. and F.T.M. using the Cochrane risk-of-bias assessment tool^45^ and the Jadad scale^46^, with papers achieving a score of less than 3 being considered low quality. Sensitivity analysis was performed based on study quality, chronology, type of resection, and whether antibiotic regimens had anaerobic and aerobic coverage consistent with contemporary guidelines.

## Results

### Literature search and clinical characteristics

**Fig. 1:**
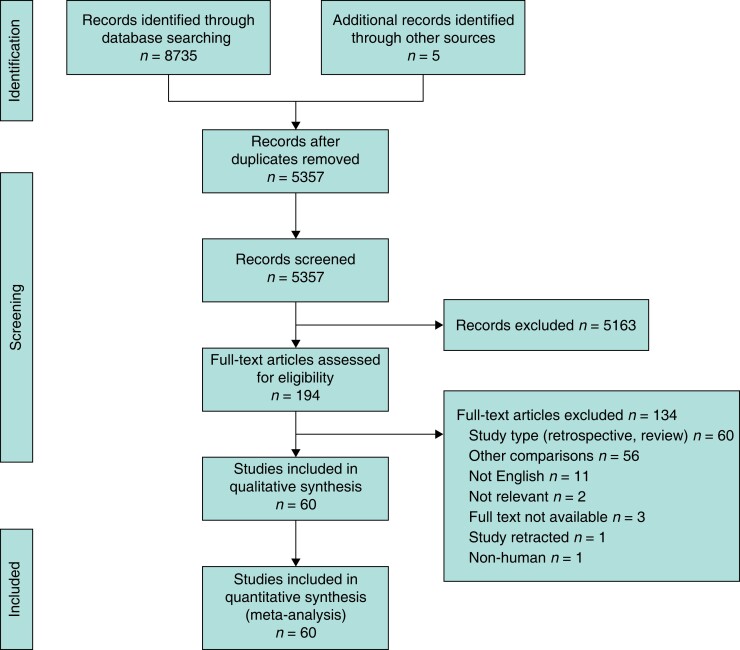
PRISMA flow diagram outlining the systematic search

From the 8735 studies identified, 60 RCTs including 16 314 patients were included (*Fig. 1*)^20–24,26,27,30,47–99^. A summary of the details of all included RCTs is included in *Table S1*. Study publication dates ranged from 1979 to 2022. Of the 60 RCTs included in this analysis, 17 studies (28 per cent) were specific to cancer resections, seven studies (12 per cent) were specific to left-sided/rectal anastomoses, and three studies (5 per cent) were specific to a laparoscopic approach. Of the 16 314 patients, 3465 (21.2 per cent) had IAB alone, 5268 (32.3 per cent) had IAB + MBP, 1710 (10.5 per cent) had IAB + OAB, 4183 (25.6 per cent) had IAB + OAB + MBP, 262 (1.6 per cent) had IAB + enemas (EN), and 1426 (8.7 per cent) had OAB + MBP.

### Primary outcomes

#### Total SSI

**Fig. 2:**
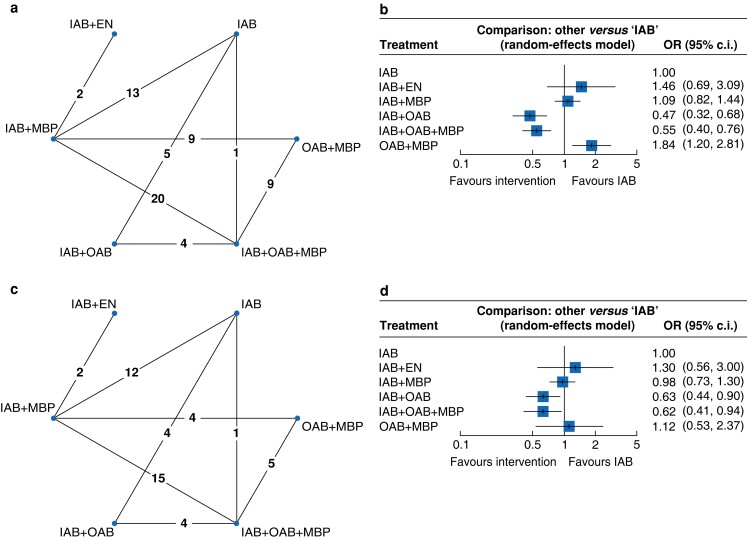
Network plots (*n* = number of studies) and forest plots for total surgical site infection and anastomotic leak **a** Network plot for total surgical site infection. **b** Forest plot for total surgical site infection. **c** Network plot for anastomotic leak. **d** Forest plot for anastomotic leak. Forest plots compare different bowel preparation methods against intravenous antibiotics. IAB, intravenous antibiotics; EN, enemas; MBP, mechanical bowel preparation; OAB, oral antibiotics.

All studies assessed SSI (*Fig. 2a*), with an overall SSI rate of 10.9 per cent. SSI rates were lowest in the IAB + OAB + MBP (7.5 per cent) and IAB + OAB (7.6 per cent) groups, compared with IAB (12.2 per cent), IAB + MBP (12.8 per cent), IAB + EN (14.1 per cent), and OAB + MBP (14.6 per cent). Using the IAB group as a baseline comparator, NMA demonstrated a statistically significant reduction in risk of total SSI with IAB + OAB (OR 0.47 (95 per cent c.i. 0.32 to 0.68)) and IAB + OAB + MBP (OR 0.55 (95 per cent c.i. 0.40 to 0.76)) (*Fig. 2b*). The SSI rate was higher in the OAB + MBP group (OR 1.84 (95 per cent c.i. 1.20 to 2.81)) compared with the IAB group. There was no significant difference with regards to total SSI rates between the IAB group and the other compared groups. League ranking tables showed IAB + OAB to be the best ranked treatment in terms of reducing the total SSI rate (*Fig. S2a*).

#### Anastomotic leak

A total of 47 studies reported AL^20–24,26,27,30,47–58,60–64,66,67,69,71,73–76,78,80,81,83–87,90–92,94,97–99^, including 13 912 patients, with an overall leak rate of 3.6 per cent (*Figure 2c*). AL rates were 1.9 per cent with IAB + OAB + MBP, 2.6 per cent with OAB + MBP, 3.5 per cent with IAB + OAB, 4.1 per cent with IAB + MBP, 5.0 per cent with IAB, and 6.1 per cent with IAB + EN. NMA showed a reduction in AL with IAB + OAB + MBP (OR 0.62 (95 per cent c.i. 0.41 to 0.94)) and IAB + OAB (OR 0.63 (95 per cent c.i. 0.44 to 0.90)) compared with IAB alone (*Fig. 2d*). There were no significant differences between the IAB group and the other compared groups. League ranking tables showed IAB + OAB + MBP to be the best treatment option, followed by IAB + OAB (*Fig. S2b*).

### Secondary outcomes

#### Superficial SSI

**Fig. 3:**
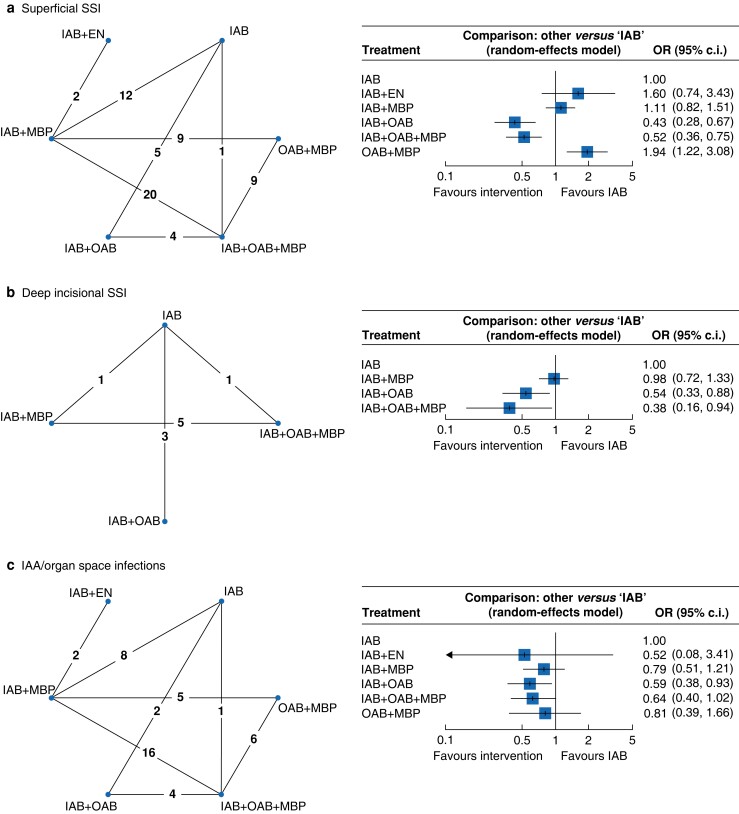
Network plots (*n* = number of studies) and forest plots for superficial surgical site infection, deep incisional surgical site infection, and intra-abdominal abscess/organ space infection **a** Network plot and forest plot for superficial surgical site infection. **b** Network plot and forest plot for deep incisional surgical site infection. **c** Network plot and forest plot for intra-abdominal abscess/organ space infection. Forest plots compare different bowel preparation methods against intravenous antibiotics. SSI, surgical site infection; IAB, intravenous antibiotics; EN, enemas; MBP, mechanical bowel preparation; OAB, oral antibiotics; IAA, intra-abdominal abscess.

All studies assessed superficial SSI (16 289 patients). The overall superficial SSI rate was 8.0 per cent and rates were lowest in the IAB + OAB (4.0 per cent) and IAB + OAB + MBP (5.2 per cent) groups, followed by IAB (7.7 per cent), IAB + MBP (10.2 per cent), OAB + MBP (12.8 per cent), and IAB + EN (13.4 per cent). NMA demonstrated a significant reduction in superficial SSI risk with IAB + OAB (OR 0.43 (95 per cent c.i. 0.28 to 0.67)) and IAB + OAB + MBP (OR 0.52 (95 per cent c.i. 0.36 to 0.75)) when compared with IAB alone, with an increased superficial SSI risk with OAB + MBP (OR 1.94 (95 per cent c.i. 1.22 to 3.08)) (*Fig. 3a*).

#### Deep incisional SSI

Ten RCTs^20,47,49–52,54,58,61,65^ assessed for deep incisional SSI (4893 patients), with an overall rate of 6.0 per cent. The rate was lowest in the IAB + OAB + MBP group (0.7 per cent), followed by IAB + OAB (3.6 per cent), IAB + MBP (7.2 per cent), and IAB alone (9.1 per cent). In comparison with IAB alone, NMA demonstrated a reduction in deep incisional SSI risk with IAB + OAB + MBP (OR 0.38 (95 per cent c.i. 0.16 to 0.94)) and IAB + OAB (OR 0.54 (95 per cent c.i. 0.33 to 0.88)) (*Fig. 3b*).

#### IAA/organ space infection

IAA/organ space infection was assessed in 47 RCTs (14 327 patients)^20,22–24,26,27,30,47,49,50,52–54,56,58,59,61–67,69–76,79,80,83–91,93–97^, with an overall rate of 2.6 per cent. IAA/organ space infection rates were lower across all treatment arms in comparison with IAB alone, with a statistically significant reduction in the IAB + OAB group (OR 0.59 (95 per cent c.i. 0.38 to 0.93)) (*Fig. 3c*). The rate was lowest in the IAB + EN group (0.8 per cent), followed by OAB + MBP (2.1 per cent), IAB + OAB + MBP (2.2 per cent), IAB + OAB (2.5 per cent), IAB + MBP (2.5 per cent), and IAB alone (3.7 per cent). IAB + OAB ranked as the best treatment option, followed by IAB + OAB + MBP, IAB + EN, IAB + MBP, OAB + MBP, and IAB alone (*Fig. S2e*).

There were no statistically significant differences between treatment options for ileus (5535 patients, 16 RCTs), return to theatre (6757 patients, 17 RCTs), respiratory tract infection (9218 patients, 22 RCTs), urinary tract infection (8884 patients, 23 RCTs), 30-day mortality rate (12350 patients, 38 RCTs), CDI (3854 patients, 13 RCTs), length of stay (8484 patients, 26 RCTs), or side effects of antibiotic therapy or bowel preparation (4461 patients, 12 RCTs) (*Fig. S3*).

### Risk-of-bias assessment

Risk-of-bias assessment demonstrated an overall low–moderate risk of bias (*Figs S4, S5*). RCT methodological quality assessment using the Jadad scale (*Table S2*) deemed 36 studies (60 per cent) to be of good quality. Participant blinding was not possible during studies that assessed MBP, contributing to a higher risk of performance bias, particularly in these trials.

### Sensitivity analysis

#### Study quality—Jadad score greater than or equal to 3

Analysis of RCTs with a Jadad score greater than or equal to 3 included 36 RCTs and 11 382 patients (*Fig. S6a*). Observed significant differences remained, with significant reductions in SSI and AL rates with IAB + OAB (OR 0.43 (95 per cent c.i. 0.30 to 0.63) and OR 0.62 (95 per cent c.i. 0.43 to 0.90) respectively) and IAB + OAB + MBP (OR 0.58 (95 per cent c.i. 0.41 to 0.82) and OR 0.56 (95 per cent c.i. 0.34 to 0.92) respectively).

#### Chronology—RCTs published after 2000

Sensitivity analysis was performed for all RCTs after 2000 (12 455 patients and 38 RCTs). This demonstrated similar results to the overall primary outcome analysis, with significant reductions in SSI and AL rates in the IAB + OAB (OR 0.46 (95 per cent c.i. 0.33 to 0.64) and OR 0.62 (95 per cent c.i. 0.43 to 0.89) respectively) and IAB + OAB + MBP (OR 0.52 (95 per cent c.i. 0.38 to 0.70) and OR 0.56 (95 per cent c.i. 0.36 to 0.89) respectively) treatment arms (*Fig. S6b*).

#### Left-sided/rectal anastomoses

Analysis of RCTs specific to left-sided/rectal anastomoses included eight RCTs and 1304 patients. There was a trend toward lower AL rates in the IAB + OAB and IAB + OAB + MBP groups (OR 0.20 (95 per cent c.i. 0.02 to 2.21) and OR 0.37 (95 per cent c.i. 0.10 to 1.40) respectively), with a statistically significant reduction in total SSI rate in the IAB + OAB + MBP group (OR 0.39 (95 per cent c.i. 0.16 to 0.95)) (*Fig. S6c*).

#### Adequacy of antibiotic coverage

A total of 28 RCTs (8229 patients) were found to utilize IAB with both aerobic and anaerobic coverage consistent with latest guidance. Results for total SSI were similar to the primary analysis, with lower SSI rates in the IAB + OAB and IAB + OAB + MBP groups (OR 0.40 (95 per cent c.i. 0.29 to 0.57) and OR 0.45 (95 per cent c.i. 0.31 to 0.66) respectively). A significant reduction in AL rate was noted in the IAB + OAB group (OR 0.63 (95 per cent c.i. 0.44 to 0.91)) (*Fig. S6d*).

## Discussion

Controversy exists over the optimal bowel preparation strategy in elective colorectal surgery, with previous European studies demonstrating low utilization of OAB (10–16.8 per cent)^36,100^. This NMA comparing different bowel preparation strategies consists solely of RCT data, including 60 RCTs and 16 289 patients. The advantage of employing NMA methodology in this analysis is that it facilitates simultaneous direct and indirect comparisons of greater than two strategies, providing more insightful results^101,102^. The most important findings were a significant reduction in risk of overall SSI and AL with strategies consisting of a combined IAB and OAB bowel preparation (that is IAB + OAB and IAB + OAB + MBP). Interestingly, the addition of MBP did not confer a statistically significant advantage over IAB + OAB alone in the overall analysis. These findings support the importance of the intestinal microbiome on anastomotic wound healing and provide strong evidence that selective antibiotic decontamination of the gastrointestinal tract with combined IAB + OAB should represent the standard of care for patients undergoing elective colorectal resection.

This NMA demonstrated a statistically significant reduction in overall SSI rates with OAB use. These findings correspond with two recently published meta-analyses^103,104^, with both showing a reduction in SSI rates with the use of preoperative OAB, and one study reporting a reduction in AL rates with OAB^104^. These data suggest that while prophylactic IAB at induction reduce the microbial burden at surgical sites through bactericidal effects, OAB may play a role in further reducing the SSI risk by eliminating the colonic mucosa-related microflora^12,13^.

The most significant finding of this NMA is a significant reduction in AL rate with the addition of OAB to IAB. The results of this NMA add to a growing body of evidence that the addition of OAB therapy reduces AL rates compared with IAB alone, and further supports the argument for preoperative OAB in elective colorectal surgery.

Historically, mechanical cleansing was thought to be among the most important factors influencing outcomes in colorectal surgery^105^. Today, the use of preoperative MBP is still commonly practiced^100,106,107^ and widely recommended^35^. The current study found no clear advantage from the addition of MBP, which contrasts with results of several previous studies, including previous large retrospective cohort studies of the ACS NSQIP colectomy database^31–33,108^, which favoured combined MBP + OAB. These findings support recent National Institute for Health and Care Excellence (NICE) and WHO guidelines, which advise that MBP alone (in the absence of OAB) should not be used to reduce SSI risk^10,109^. This brings into question the true efficacy of and necessity for MBP, which has been shown to cause alterations in the colonic mucosa and inflammatory changes in the lamina propria^110–112^, which may result in increased risk of bacterial translocation^113–115^. Furthermore, attempted but ineffective bowel preparation may lead to difficulties controlling large volumes of liquid stool, increasing the risk of faecal spillage and contamination^21^. Nevertheless, ranking tables suggest that MBP may still confer a potential advantage over IAB + OAB, with regards to AL risk, and sensitivity analysis suggests that MBP may play a role in left-sided/rectal resections. Overall, the proportion of benefit attributable to each of MBP and OAB remains uncertain, and ongoing large-volume RCTs^116^, including the ORALEV2 (NCT04161599)^117^, MOBILE2 (NCT04281667)^118^, and SELDDEC^119^ studies, may shed more light on this long-standing debate.

Some authors have advocated against the use of OAB, due to a theoretical higher risk of CDI^17^, yet this NMA did not identify a significant difference in CDI risk with OAB. Currently, there is limited evidence to suggest a significant causal relationship between OAB and CDI in the setting of colorectal surgery^64,120^, with some studies even suggesting a lower rate of *C. difficile* colitis with OAB^33,121,122^. Overall, the benefits of OAB outweigh the theoretical low risk of post-colectomy *C. difficile* colitis, and OAB should not be omitted over concerns regarding potential CDI.

While the strength of a NMA methodology is its ability to allow for simultaneous comparisons between several treatment options both directly and indirectly, there are pertinent limitations of such techniques^101^. As with any meta-analysis, clinical and methodological heterogeneity will exist given the differences in patient, antibiotic, and bowel preparation selection within the individual included studies. The results should also be interpreted with caution given the differences between sizes of each treatment arm—the majority of patients had IAB, IAB + MBP, or IAB + OAB + MBP; IAB + OAB represented 10.5 per cent of patients, with fewer studies reporting outcomes for this antimicrobial strategy. The methods of bowel preparation and types of OAB selection were also not distinguished in the analysis. OAB and MBP strategies varied, with noted heterogeneity between studies. Where OAB were utilized, most included non-absorbable aminoglycosides (for example neomycin and kanamycin). Many contemporary studies also utilized polyethylene glycol, sodium picosulfate, or sodium phosphate solutions for MBP. The current methodology did not differentiate between resections for benign and malignant disease or those in receipt of preoperative chemoradiation, laparoscopic *versus* open procedures, operative time, stapled *versus* handsewn anastomoses, and the presence or absence of a diverting stoma—all of which may be important confounding factors that may ultimately influence outcomes.^123–125^

These data demonstrate that OAB produce a significant reduction in SSI and AL, and provide high-level evidence to recommend the implementation of preoperative OAB as the standard of care in elective colorectal resection. There is less clarity surrounding the role of MBP, and the authors eagerly anticipate the results of the large ongoing, multicentre RCTs that may provide a more conclusive answer to this question.^117,118^

## Supplementary Material

zrad040_Supplementary_Data

## Data Availability

All data are included in the manuscript, referenced articles, or the supplementary material.
