# Supplementary material for: Mechanical bowel preparation and antibiotics in elective colorectal surgery: network meta-analysis
Source: BJS Open. 2023 May 31;7(3):zrad040. doi: 10.1093/bjsopen/zrad040 (PMC10231808; doi:10.1093/bjsopen/zrad040)
Supplement: zrad040_Supplementary_Data [file zrad040_supplementary_data.zip › Supplementary Materials - Updated References.docx]

**Mechanical Bowel Preparation and Antibiotics in Elective Colorectal Surgery: Systematic Review and Network Meta-analysis of Randomised Clinical Trials**

Jonavan Tan MB MRCS^1^, Éanna J. Ryan MD MRCS^1^, Matthew G. Davey MRCS PhD^2^, Fiachra T. McHugh MCh^1^, Ben Creavin MD MRCS^1^, Maria C. Whelan MCh FRCS^1^, Michael E. Kelly MCh FRCS^1^, Paul C. Neary MD FRCS^1,3^, Dara O. Kavanagh MCh FRCS^1,4^, James M. O’Riordan MD FRCS^1,5^.

*^1^Department of Colorectal Surgery, Tallaght University Hospital, Tallaght, Dublin 24*

*^2^Department of Surgery, Royal College of Surgeons in Ireland, Dublin 2*

*^3^Clinical Professor of Surgery, Trinity College Dublin, College Green, Dublin 2*

*^4^Senior Lecturer, Royal College of Surgeons Ireland, 123 St Stephen's Green, Dublin 2*

*^5^Senior Lecturer, Trinity College Dublin, College Green, Dublin 2*

**Corresponding author:** Mr Éanna Ryan MB BCh BAO, MRCSI, MD (ORCID ID 0000-0003-2609-0836)

**Supplementary Materials - Index**

| **Supplementary Methods** |  |
| --- | --- |
| Search strategy | *pag. 2* |
| **Supplementary Figures and Tables** |  |
| Data from all included RCTs | *pag. 3* |
| Ranked treatment effects | *pag. 6* |
| Secondary outcomes – Network & Forest plots | *pag. 8* |
| Risk of Bias | *pag. 10* |
| Sensitivity Analysis | *pag. 12* |
|  |  |

**Supplementary Methods**

**Supplementary Figure 1. Search Strategy**

The following standard search headings and medical subject headings (MeSH) were used:

**((bowel preparation) OR antibiotic) AND colorectal surgery**

- ((("bowel s"[All Fields] OR "bowel"[All Fields] OR "intestines"[MeSH Terms] OR "intestines"[All Fields] OR "bowel"[All Fields] OR "bowels"[All Fields]) AND ("preparate"[All Fields] OR "preparates"[All Fields] OR "preparation"[All Fields] OR "preparations"[All Fields] OR "preparative"[All Fields] OR "preparatively"[All Fields] OR "prepare"[All Fields] OR "prepared"[All Fields] OR "prepares"[All Fields] OR "preparing"[All Fields])) OR ("anti bacterial agents"[Pharmacological Action] OR "anti bacterial agents"[MeSH Terms] OR ("anti bacterial"[All Fields] AND "agents"[All Fields]) OR "anti bacterial agents"[All Fields] OR "antibiotic"[All Fields] OR "antibiotics"[All Fields] OR "antibiotic s"[All Fields] OR "antibiotical"[All Fields])) AND ("colorectal surgery"[MeSH Terms] OR ("colorectal"[All Fields] AND "surgery"[All Fields]) OR "colorectal surgery"[All Fields])

**Supplementary Results**

***Supplementary Table 1. Data from the 60 RCTs included in this systematic review and network meta-analysis***. *MBP: Mechanical Bowel Preparation, OAB: Oral Antibiotics, IV: Intravenous Antibiotics, ED: Enema, PEG: polyethlene Glycol.*

***Supplementary Figure 2. Ranked Treatment Effects.*** *NMA league ranking chart of treatments regarding AL rate using indirect and direct pairwise comparisons. Treatments are ranked from best to worst along the leading diagonal. Above the leading diagonal are estimates from pairwise meta-analyses, below the leading diagonal are estimates from NMA.* *RCTs included in analysis, where not already stated in the main manuscript, are listed as references.*

1. *Total SSI* ([20-24](#_ENREF_20), [26](#_ENREF_26), [27](#_ENREF_27), [30](#_ENREF_30), [46-97](#_ENREF_46))

*
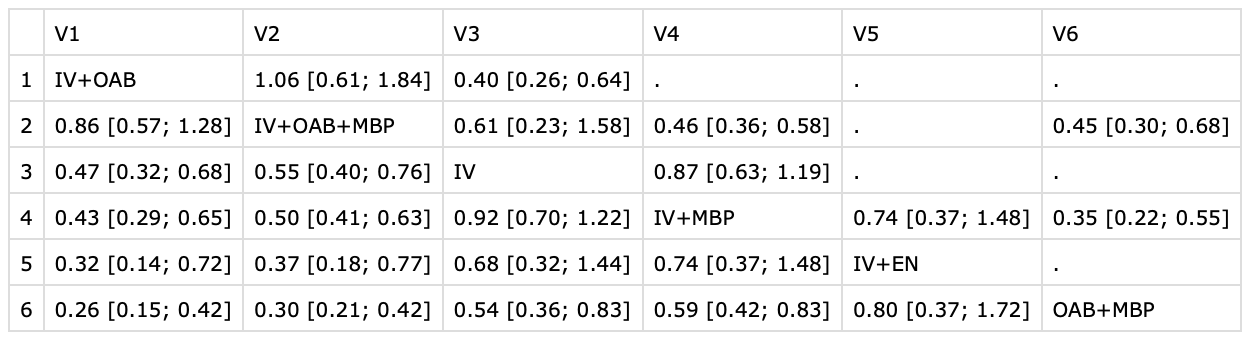
*

1. Anastomotic Leak

*
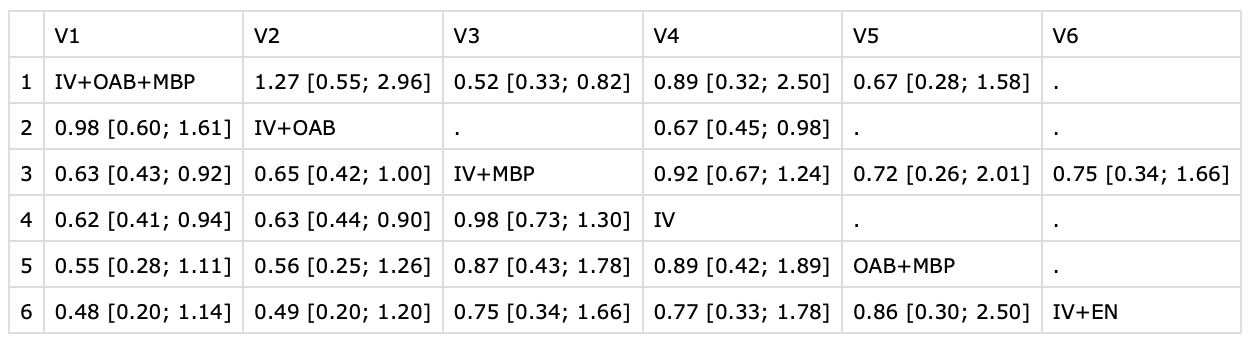
*

1. Superficial SSI


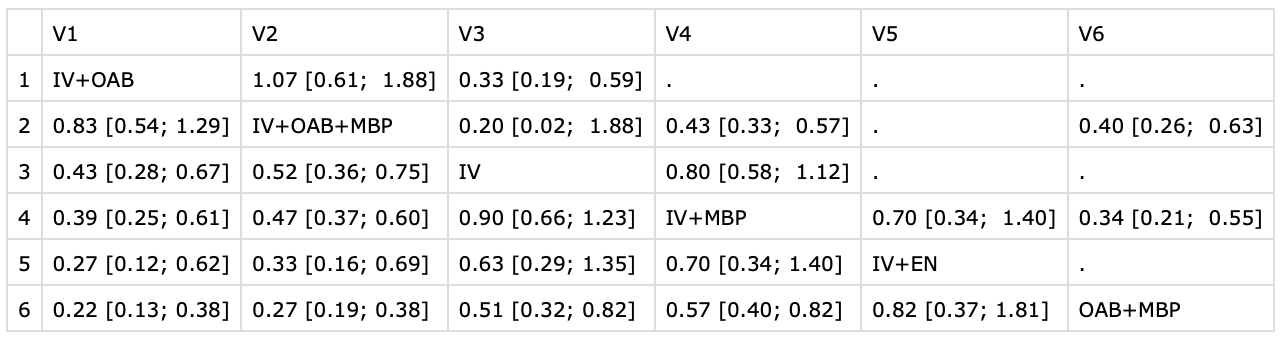


1. Deep Incisional SSI


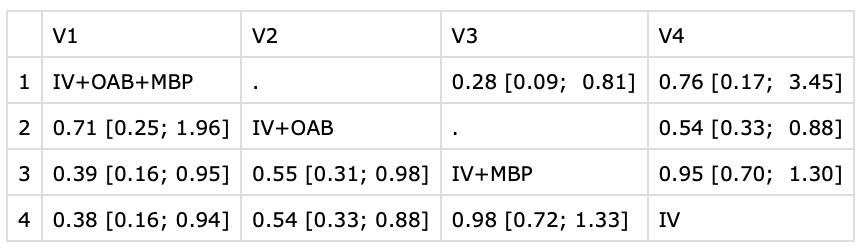


1. Organ Space Infection


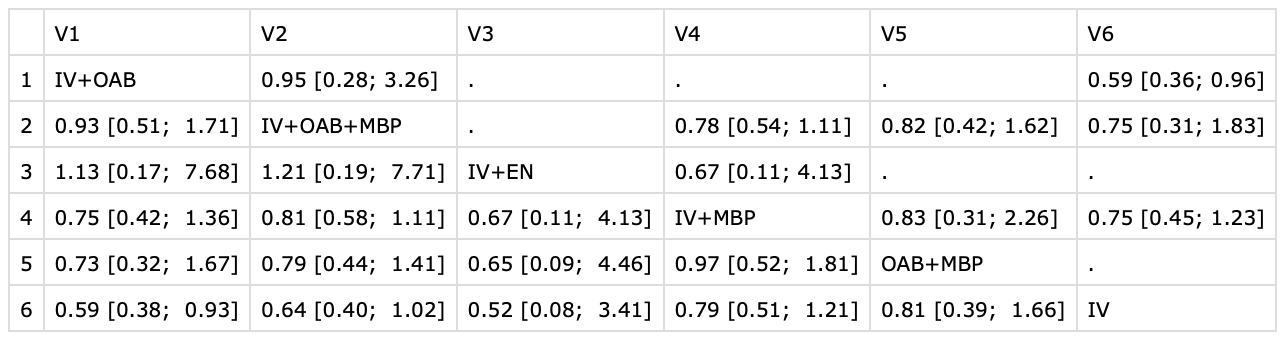


1. 30-Day Mortality ([20](#_ENREF_20), [22-24](#_ENREF_22), [26](#_ENREF_26), [27](#_ENREF_27), [30](#_ENREF_30), [46-50](#_ENREF_46), [52-54](#_ENREF_52), [56](#_ENREF_56), [59](#_ENREF_59), [61](#_ENREF_61), [62](#_ENREF_62), [66](#_ENREF_66), [68-70](#_ENREF_68), [72](#_ENREF_72), [73](#_ENREF_73), [77](#_ENREF_77), [80](#_ENREF_80), [83-87](#_ENREF_83), [89](#_ENREF_89), [90](#_ENREF_90), [92](#_ENREF_92), [94](#_ENREF_94), [96](#_ENREF_96), [97](#_ENREF_97))


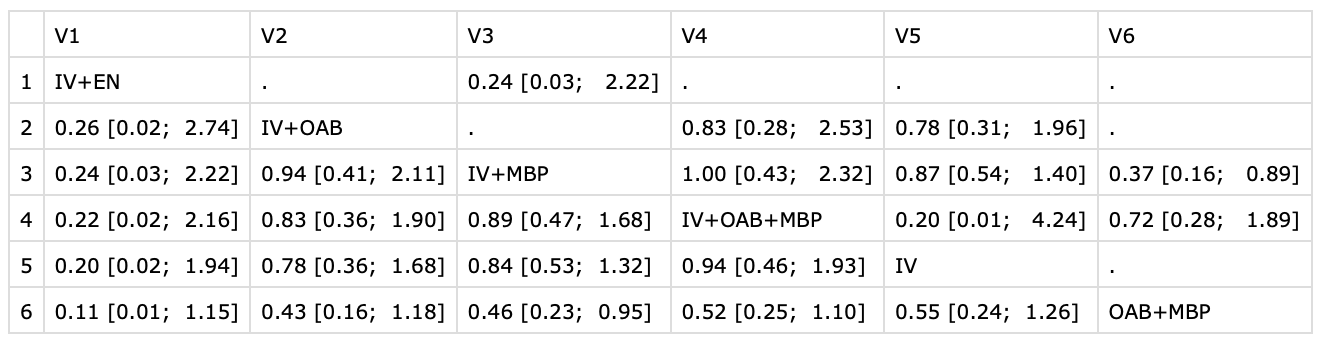


1. Return to Theatre ([20-24](#_ENREF_20), [26](#_ENREF_26), [30](#_ENREF_30), [46](#_ENREF_46), [47](#_ENREF_47), [50](#_ENREF_50), [52](#_ENREF_52), [53](#_ENREF_53), [56](#_ENREF_56), [57](#_ENREF_57), [62](#_ENREF_62), [64](#_ENREF_64), [70](#_ENREF_70))


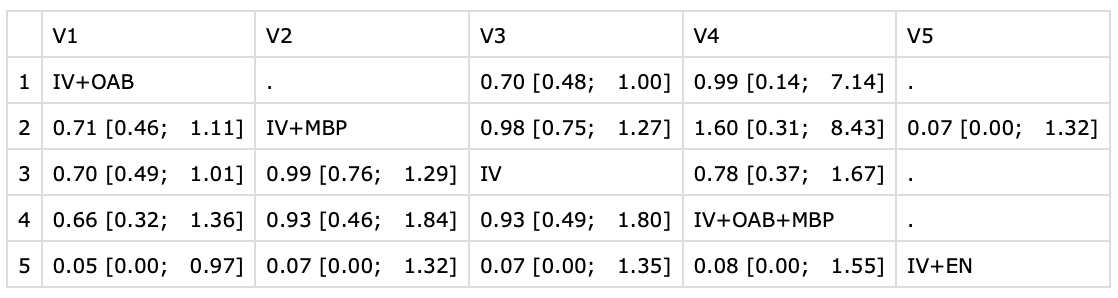


1. Length of Stay ([20-24](#_ENREF_20), [26](#_ENREF_26), [27](#_ENREF_27), [30](#_ENREF_30), [46](#_ENREF_46), [49](#_ENREF_49), [50](#_ENREF_50), [52-54](#_ENREF_52), [59](#_ENREF_59), [65-67](#_ENREF_65), [70](#_ENREF_70), [72](#_ENREF_72), [78](#_ENREF_78), [82](#_ENREF_82), [89](#_ENREF_89), [92](#_ENREF_92), [95](#_ENREF_95), [96](#_ENREF_96))


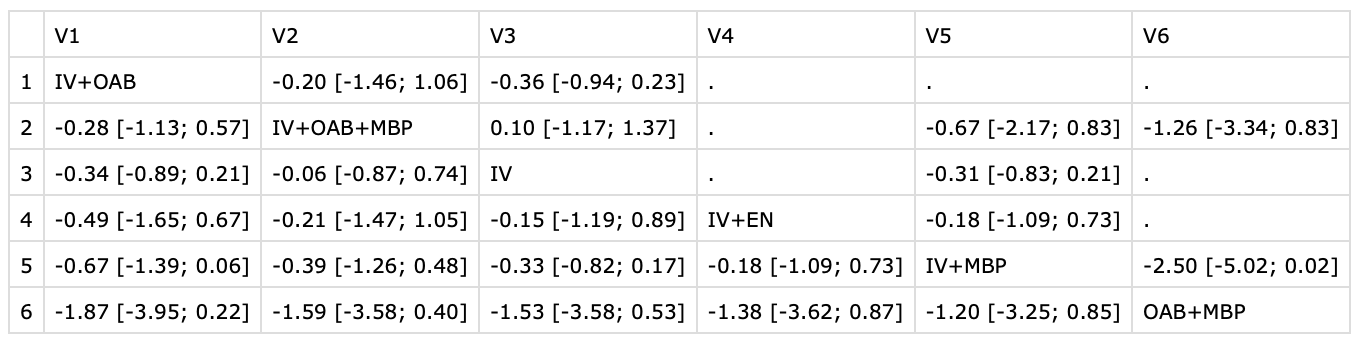


1. Ileus ([22](#_ENREF_22), [24](#_ENREF_24), [48](#_ENREF_48), [49](#_ENREF_49), [51](#_ENREF_51), [53](#_ENREF_53), [56](#_ENREF_56), [60](#_ENREF_60), [65-67](#_ENREF_65), [71](#_ENREF_71), [72](#_ENREF_72), [81](#_ENREF_81), [92](#_ENREF_92), [95](#_ENREF_95))


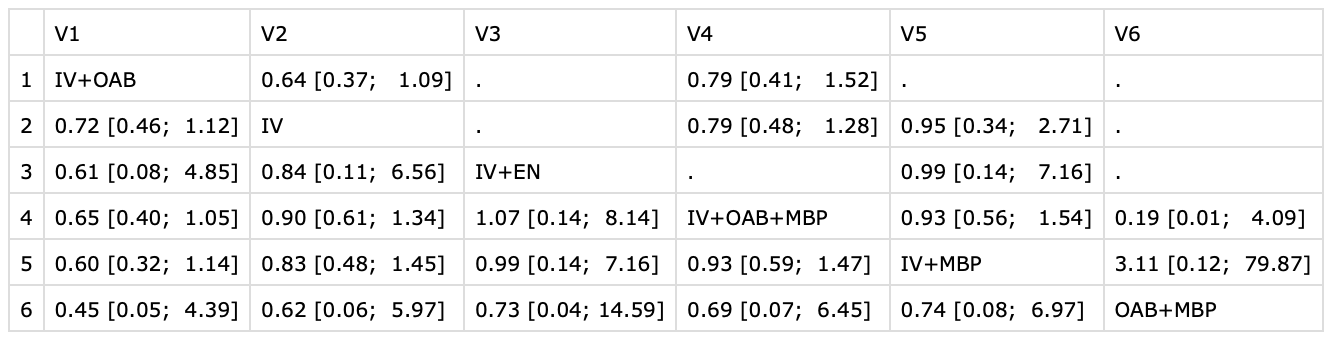


1. Side Effects of ABX / MBP ([48-50](#_ENREF_48), [53](#_ENREF_53), [56](#_ENREF_56), [58](#_ENREF_58), [64](#_ENREF_64), [69](#_ENREF_69), [79](#_ENREF_79), [85](#_ENREF_85), [88](#_ENREF_88), [90](#_ENREF_90))


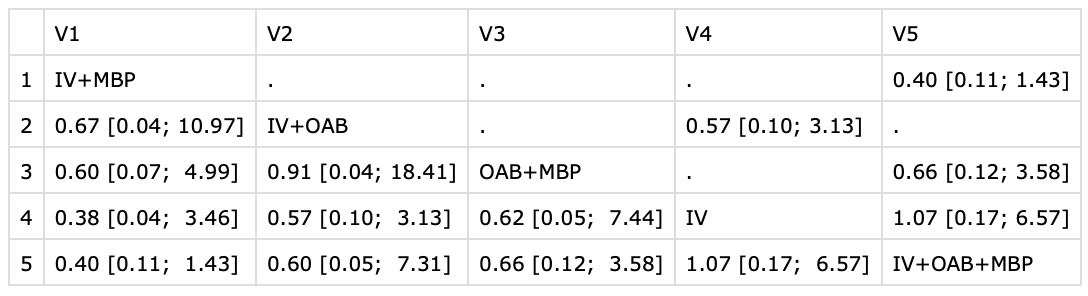


1. Respiratory Tract Infection([20](#_ENREF_20), [22](#_ENREF_22), [26](#_ENREF_26), [30](#_ENREF_30), [46](#_ENREF_46), [47](#_ENREF_47), [49](#_ENREF_49), [52](#_ENREF_52), [53](#_ENREF_53), [55-57](#_ENREF_55), [59](#_ENREF_59), [60](#_ENREF_60), [71](#_ENREF_71), [72](#_ENREF_72), [74](#_ENREF_74), [75](#_ENREF_75), [84](#_ENREF_84), [89](#_ENREF_89), [90](#_ENREF_90), [95](#_ENREF_95))


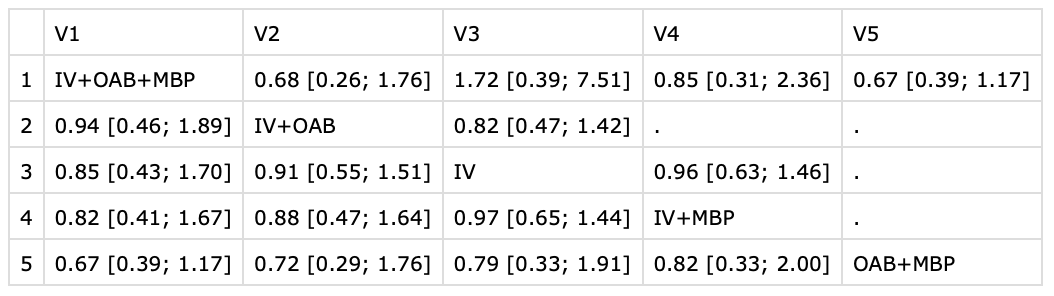


1. *Urinary Tract Infection (*[20](#_ENREF_20)*,* [22](#_ENREF_22)*,* [24](#_ENREF_24)*,* [26](#_ENREF_26)*,* [30](#_ENREF_30)*,* [47](#_ENREF_47)*,* [49](#_ENREF_49)*,* [52](#_ENREF_52)*,* [53](#_ENREF_53)*,* [55-57](#_ENREF_55)*,* [59](#_ENREF_59)*,* [60](#_ENREF_60)*,* [66](#_ENREF_66)*,* [71](#_ENREF_71)*,* [72](#_ENREF_72)*,* [75](#_ENREF_75)*,* [84](#_ENREF_84)*,* [86](#_ENREF_86)*,* [89](#_ENREF_89)*,* [90](#_ENREF_90)*,* [95](#_ENREF_95)*)*


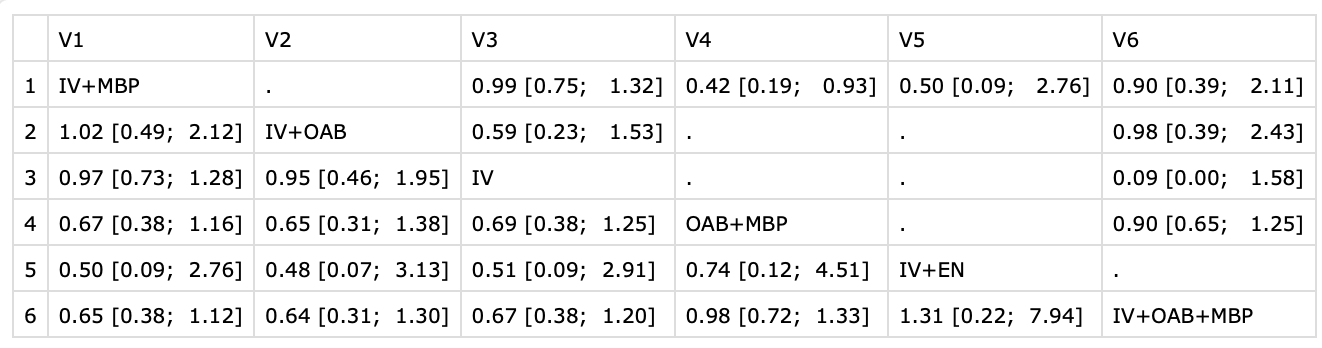


1. *Clostridium Difficile Infection (*[47](#_ENREF_47)*,* [48](#_ENREF_48)*,* [50](#_ENREF_50)*,* [53](#_ENREF_53)*,* [55-58](#_ENREF_55)*,* [60](#_ENREF_60)*,* [61](#_ENREF_61)*,* [64](#_ENREF_64)*,* [81](#_ENREF_81)*,* [97](#_ENREF_97)*)*


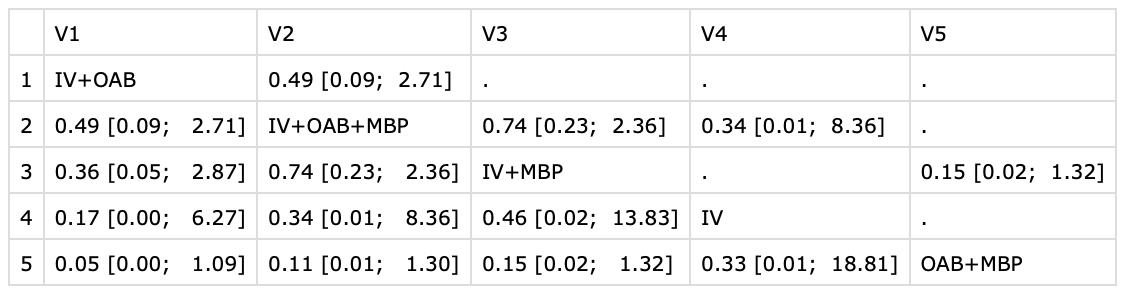


***Supplementary Figure 3. Network and Forest Plots for Remainder of Secondary Outcomes.*** *The number of studies being compared (n) are indicated on the lines of the network plot.* *Forest plots compare different bowel preparation methods against IV.*

1. *30 Day Mortality*


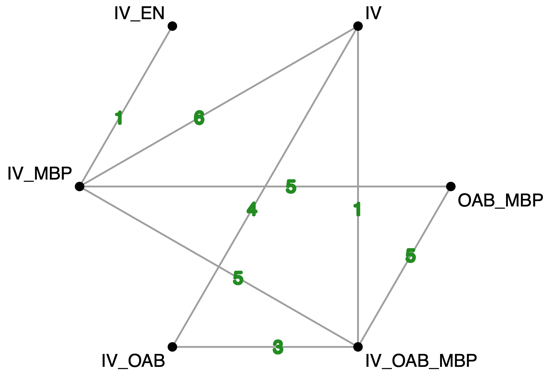

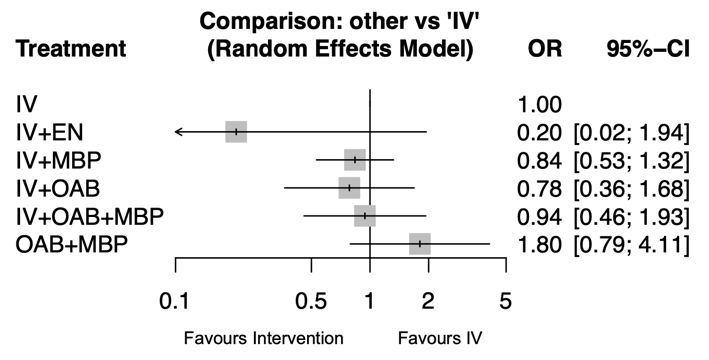


1. *Return to Theatre*


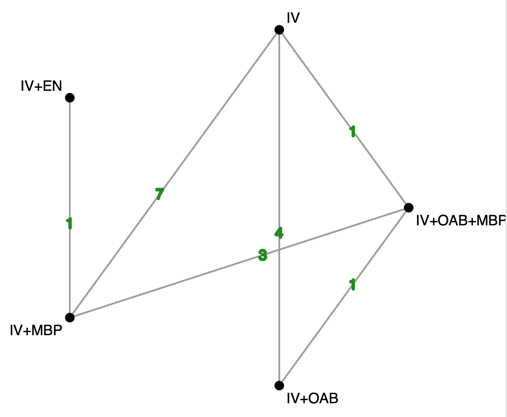

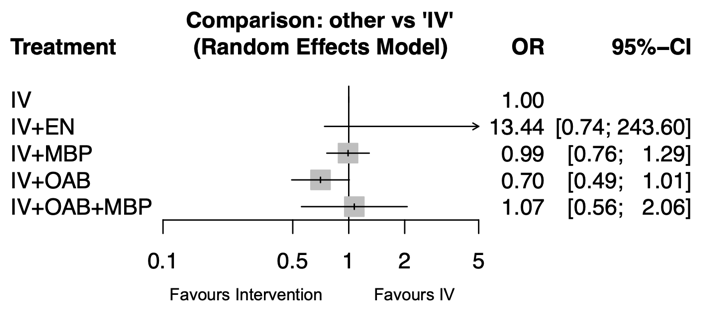


1. *Length of Stay*


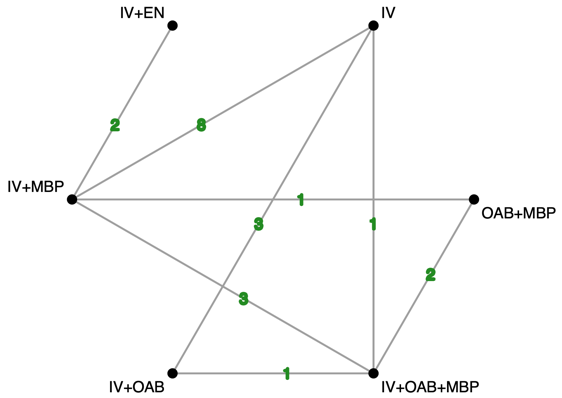

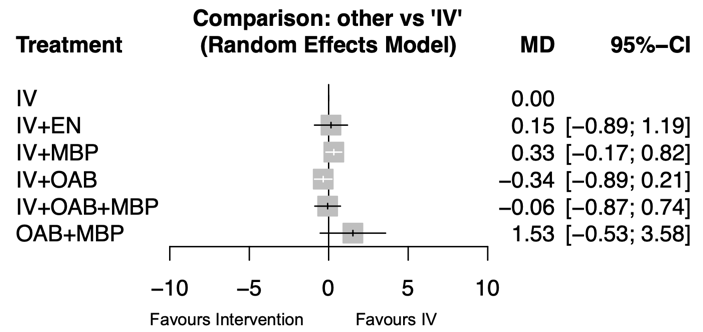


1. *Ileus*


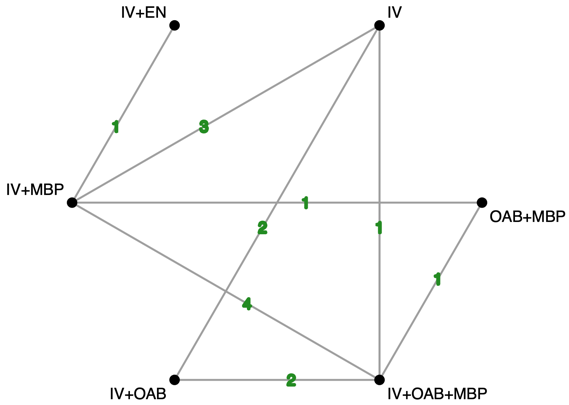

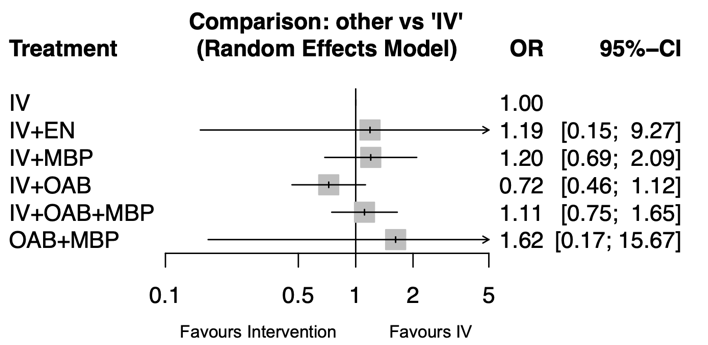


1. Side Effects of ABX / MBP


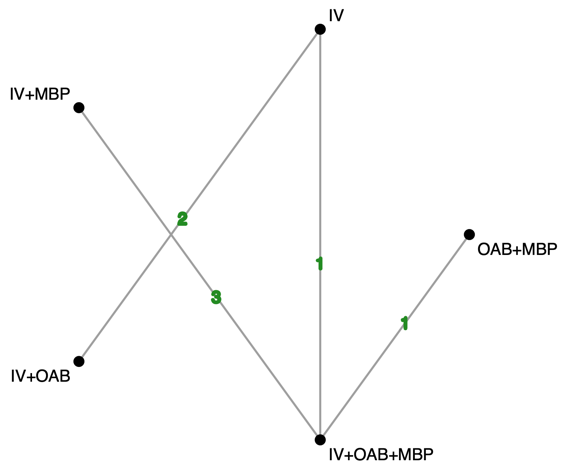

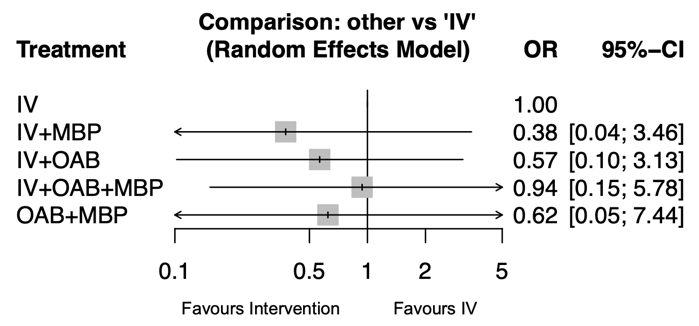


1. Respiratory Tract Infection


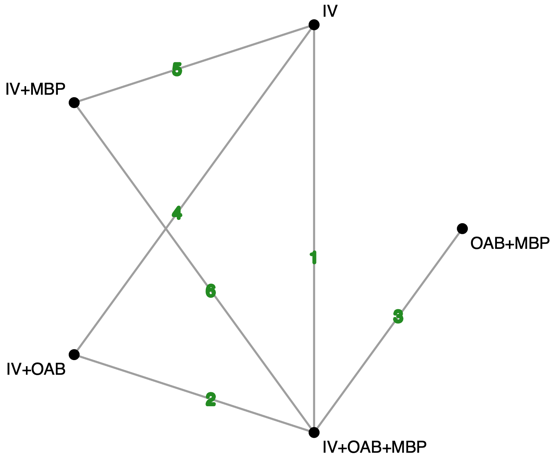

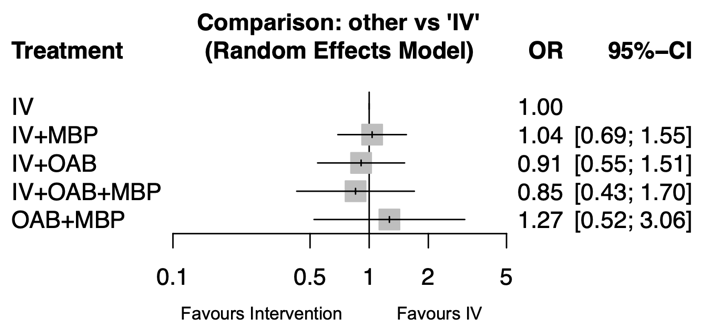


1. *Urinary Tract Infection*


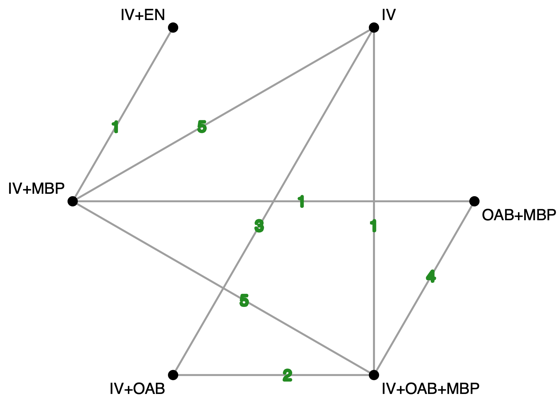

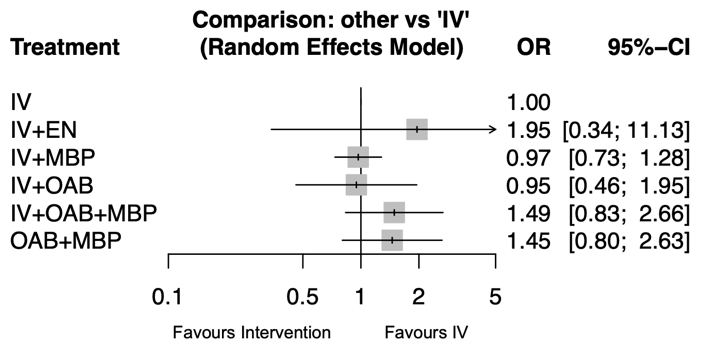


1. *Clostridium Difficile Infection*


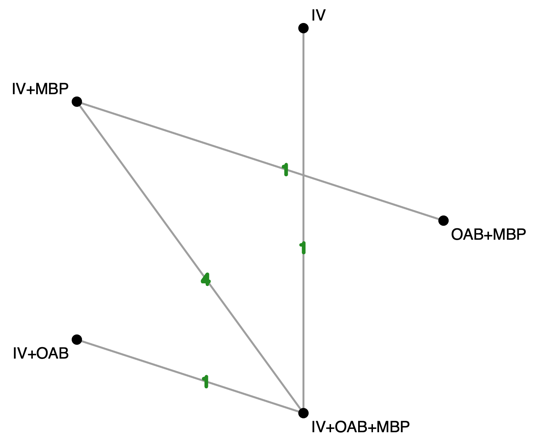

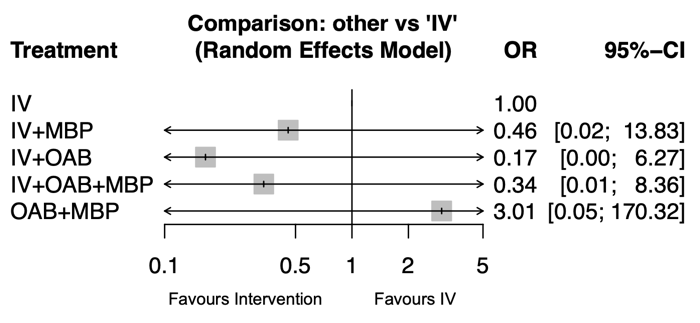


***Supplementary Figure 4. Types and risk of bias for the randomised control trials included in this study.*** *Green outcomes indicate a low risk of bias*


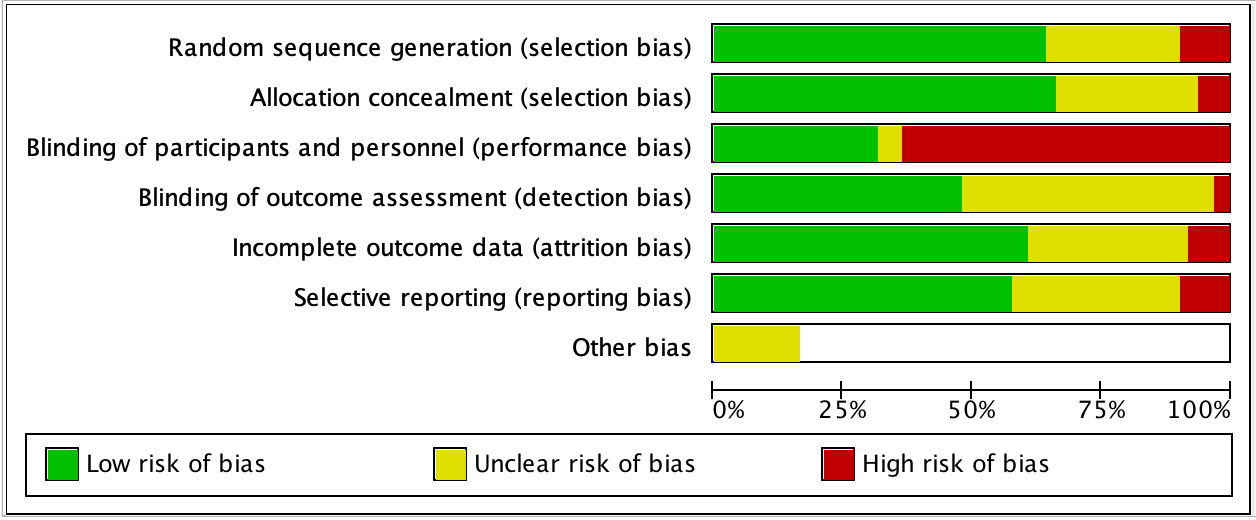

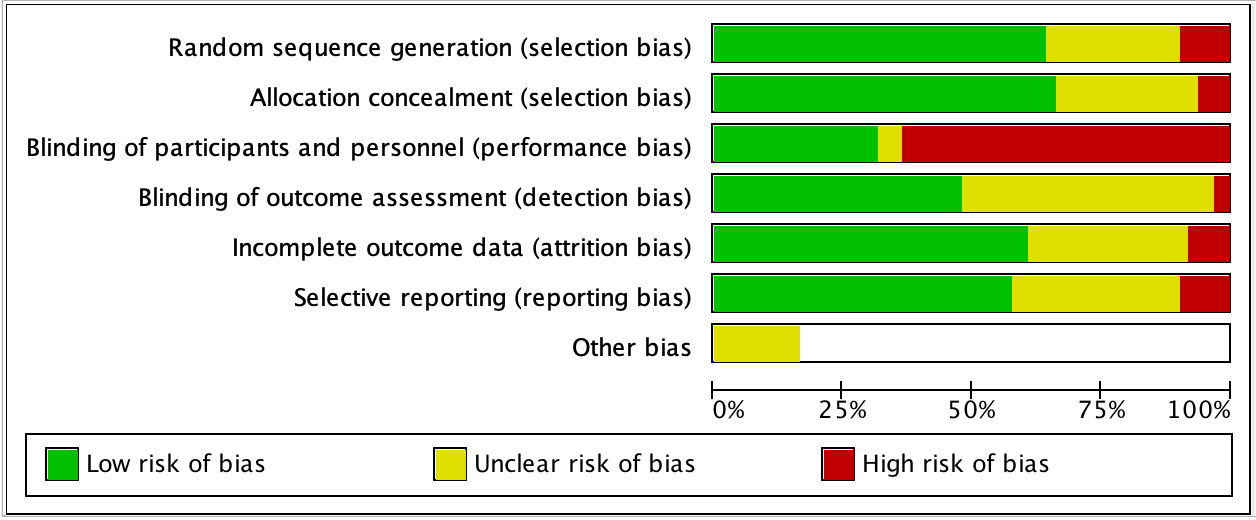


***Supplementary Figure 5. Risk of Bias for individual RCTs included in this study.*** *Green outcomes indicate low risk of bias.*


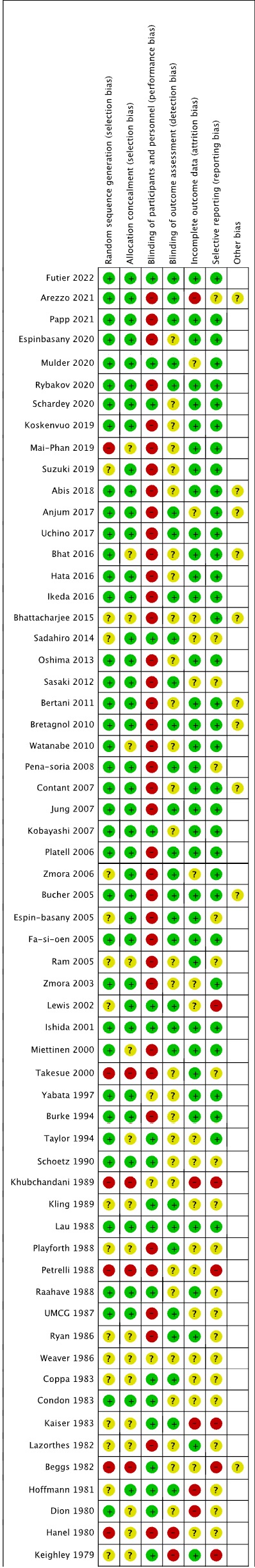


***Supplementary Table 2. Jadad score for individual RCTs included in this study.*** *Columns refer to (A) if randomisation was mentioned, (B) if randomisation was appropriate, (C) if the study was double-blinded, (D) if method of blinding was appropriate and (E) if all patients are accounted for*

***Supplementary Figure 6. Forest and Network Plots for Sensitivity Analysis.*** *Forest plots compare different bowel preparation methods against IV. The number of studies being compared (n) are indicated on the lines of the network plot.* *RCTs included in analysis are listed as references.*

1. ***Study Quality (Jadad ≥3)*** *(*[20-23](#_ENREF_20)*,* [26](#_ENREF_26)*,* [30](#_ENREF_30)*,* [46-53](#_ENREF_46)*,* [56-62](#_ENREF_56)*,* [65-70](#_ENREF_65)*,* [72-74](#_ENREF_72)*,* [76](#_ENREF_76)*,* [82](#_ENREF_82)*,* [91](#_ENREF_91)*,* [93-95](#_ENREF_93)*)*

*Anastomotic Leak*

*
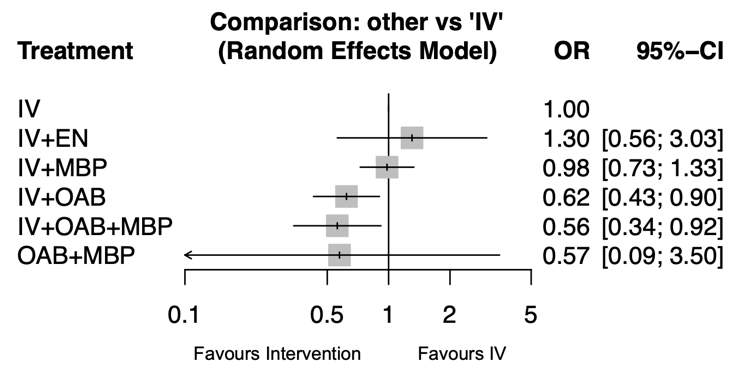

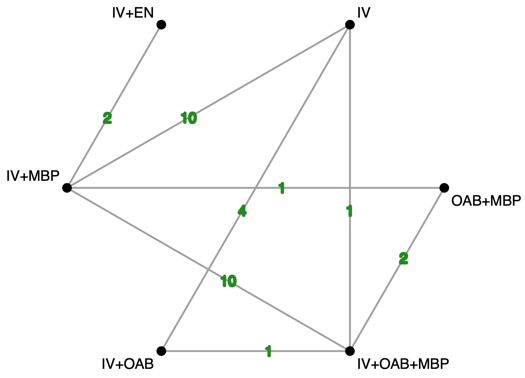
*

*Total SSI*

*
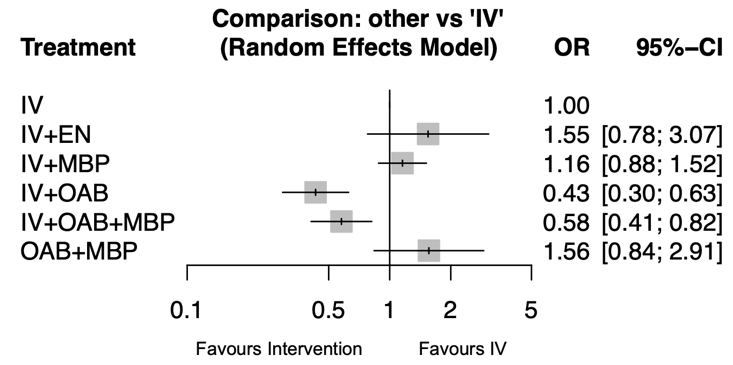

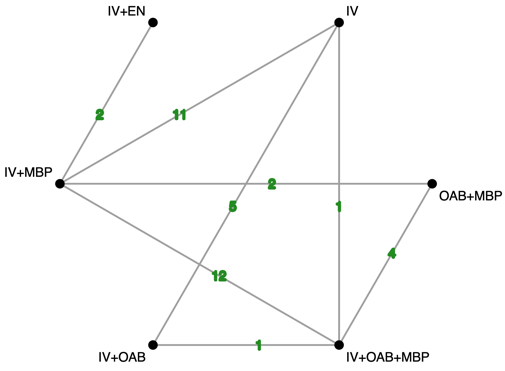
*

1. ***Chronology – RCTs after 2000*** ([20-24](#_ENREF_20), [26](#_ENREF_26), [27](#_ENREF_27), [30](#_ENREF_30), [46-75](#_ENREF_46))

*Anastomotic Leak*

*
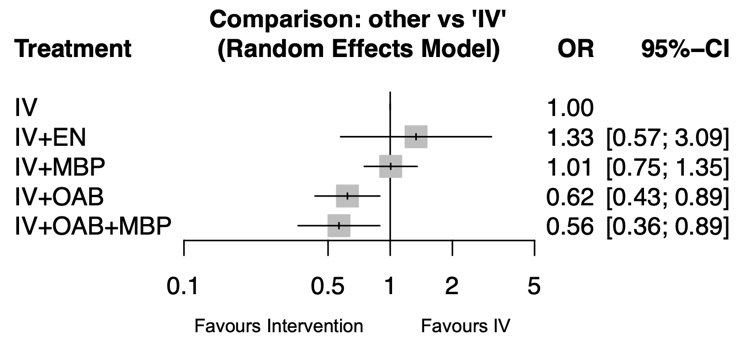

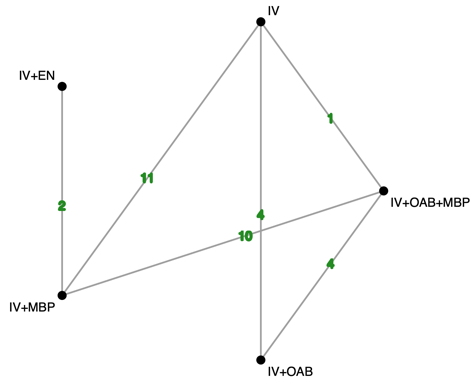
*

*Total SSI*

***
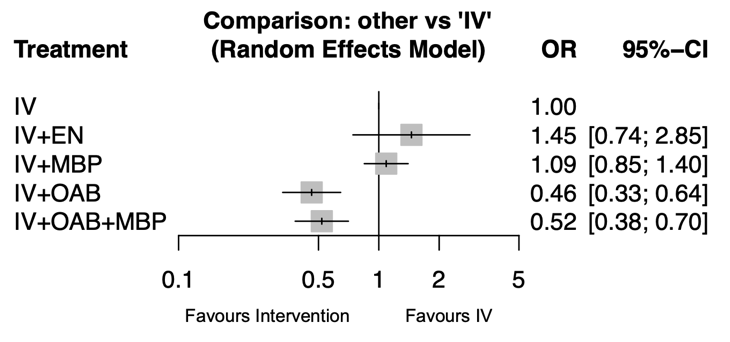

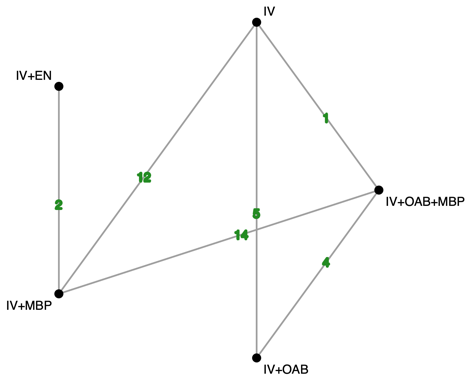
***

1. ***Left Colonic / Rectal Anastomoses*** *(*[26](#_ENREF_26)*,* [27](#_ENREF_27)*,* [30](#_ENREF_30)*,* [51](#_ENREF_51)*,* [52](#_ENREF_52)*,* [64](#_ENREF_64)*,* [77](#_ENREF_77)*,* [98](#_ENREF_98)*)*

*Anastomotic Leak*

*
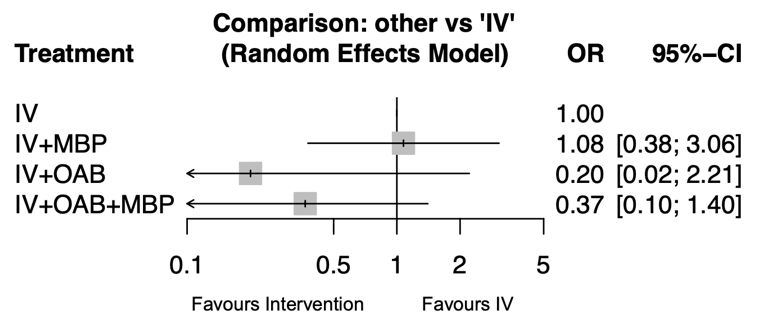

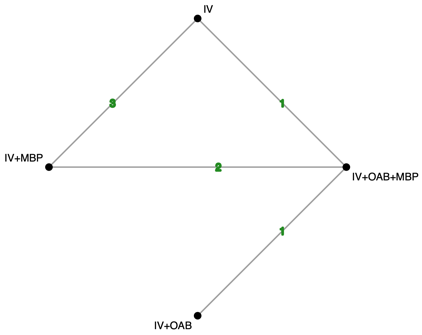
*

*Total SSI*

***
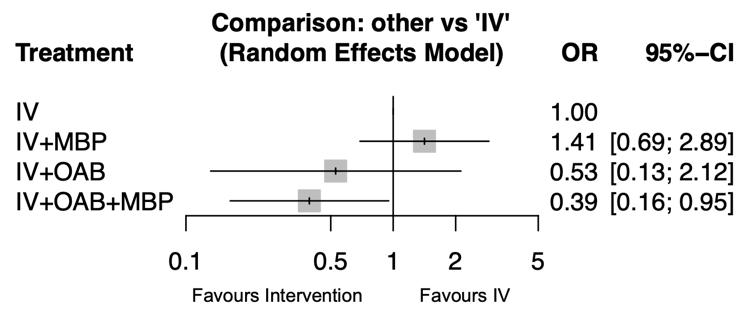

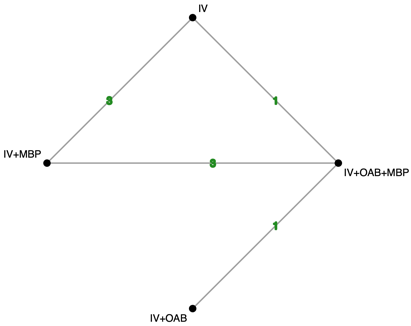
***

1. ***Adequacy of IV Antibiotic Coverage*** ([20](#_ENREF_20), [21](#_ENREF_21), [23](#_ENREF_23), [24](#_ENREF_24), [26](#_ENREF_26), [27](#_ENREF_27), [30](#_ENREF_30), [46-50](#_ENREF_46), [53](#_ENREF_53), [56](#_ENREF_56), [57](#_ENREF_57), [59](#_ENREF_59), [62](#_ENREF_62), [68](#_ENREF_68), [70](#_ENREF_70), [72](#_ENREF_72), [73](#_ENREF_73), [77](#_ENREF_77), [82](#_ENREF_82), [86](#_ENREF_86), [88](#_ENREF_88), [92](#_ENREF_92), [96](#_ENREF_96), [97](#_ENREF_97))

*Anastomotic Leak*

*
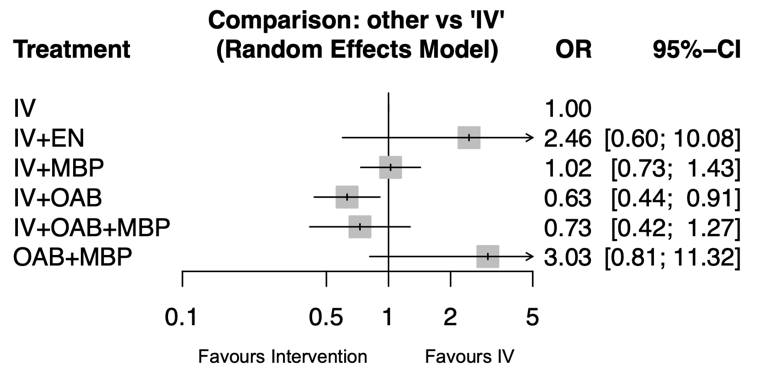

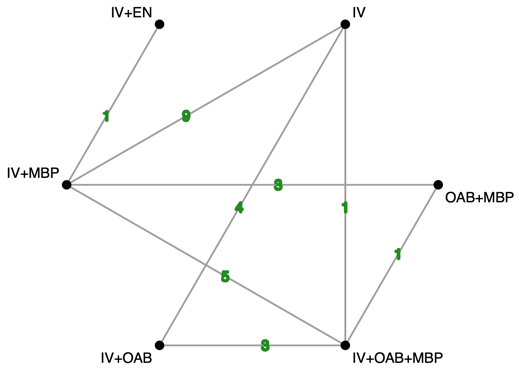
*

*Total SSI*

**
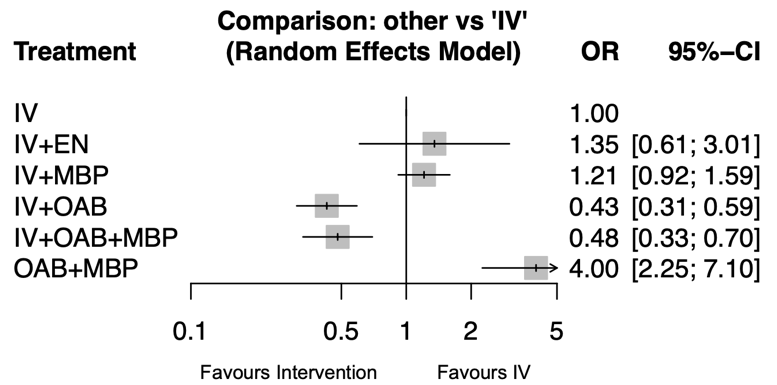

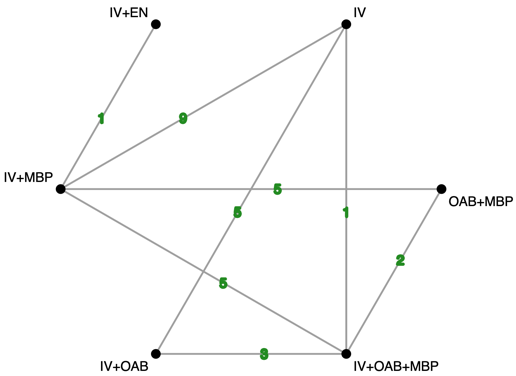
**
